# Influence of the emulsifier on nanostructure and clinical application of liquid crystalline emulsions

**DOI:** 10.1038/s41598-023-31329-w

**Published:** 2023-03-14

**Authors:** Veerawat Teeranachaideekul, Siriwat Soontaranon, Supreeya Sukhasem, Doungdaw Chantasart, Amaraporn Wongrakpanich

**Affiliations:** 1 Department of Pharmacy, Faculty of Pharmacy, Mahidol University, Bangkok, 10400 Thailand; 2 Synchrotron Light Research Institute (Public Organization), Nakhon Ratchasima, 30000 Thailand; 3 Research Project Management Group, Postharvest and Processing Research and Development Division, Department of Agriculture, Bangkok, 10900 Thailand

**Keywords:** Nanomedicine, Drug delivery, Pharmaceutics

## Abstract

Liquid crystals are appealing in pharmaceutical and cosmetic fields due to their unique structures that combine the properties of both liquid and solid states. Forming an emulsion into liquid crystals can be affected by a number of factors, including the emulsion composition and temperature. Changing the types and concentrations of surfactants could be another factor that affects liquid crystals. Currently, most liquid crystal research focuses on the nanostructure of liquid crystal systems without evaluating the efficacy of liquid crystals clinically. In this study, liquid crystalline emulsions made from camellia seed oil with four different surfactants (Olivem 1000, Polyaquol-2W, Nikkomulese LC, and Lecinol S-10 with Tween 80) were created. The liquid crystal emulsions were formulated in the form of oil-in-water (o/w) emulsions with *Camellia oleifera* seed oil serving as the main ingredient in the oil phase (10% w/w). All formulations exhibited liquid crystal characteristics with lamellar structures as determined by the polarized light microscopy and small-angle X-ray scattering with supporting data of the nanostructure from wide-angle X-ray scattering and differential scanning calorimetry (DSC). They all showed good stability under normal (room temperature) and accelerated conditions (4 °C and 40 °C) in long-term storage (6 months). Using the reconstructed human epidermis as a skin model, all formulations did not cause skin irritation. In the clinical trial, all formulations were able to reduce transepidermal water loss (TEWL) and increase skin hydration immediately after application. This lasted at least 10 h. All formulations showed distinct Maltese crosses under the polarized light microscope with a positive result for liquid crystals in wide angle X-ray scattering (WAXS) and small angle X-ray scattering (SAXS) methods. Moreover, among all formulations tested, Formulation D, which contained Lecinol S-10 and Tween 80 as emulsifiers, showed the most robust interaction between the surfactant and water molecules in the lamellar structure under DSC. The formulation was stable in long-term normal and accelerated conditions. Above all, Formulation D, which was formulated with Lecinol S-10 with Tween 80, had the best clinical result, was nonirritating to the skin, and can be used as a cream base in the pharmaceutical and cosmeceutical sectors.

## Introduction

Liquid crystals are a special state of matter that combines the properties of both the liquid and solid states. Liquid crystals have the crystal order of solids and the flow characteristics of liquids^1^. Liquid crystals have different architectures, from hexagonal, cubic, reverse hexagonal, and lamellar^2^. The system can generally be formed using self-assembled surfactants or lipids in aqueous media as an oil-in-water (o/w) emulsion. Liquid crystals are attracting the attention of many pharmaceutical and cosmetic researchers due to their unique microstructures and physicochemical properties. Yamada et al.^3^ prepared liquid crystals in the cubic and reversed hexagonal phases that can enhance calcein permeation through the skin. Lee et al.^4^ formulated liquid crystals as nanoparticles for oral drug delivery. There are reports of various liquid crystal formulations loaded with actives, such as azelaic acid^5^ and mulberry stem extract^6^.

Among all types of liquid crystals, lamellar crystals are the most attractive since the lamellar structure can resemble the skin structure. Lamellar crystals can entrap a wide range of lipophilic and hydrophilic drug molecules within the molecular arrangement of lipid bilayers and aqueous channels of the lamellar phase, respectively. It is possible that the liquid crystal structure can improve the skin moisturizing property^7^. Iwai et al.^8^ formulated liquid crystals in a lamella form using a synthesized pseudoceramide that showed better permeability, skin hydration, and skin occlusion when compared to regular emulsions. Liquid crystals could act as a skin barrier that can reduce transepidermal water loss (TEWL) and increase skin hydration^9^.

*Camellia oleifera* Abel. is one of the four major sources of edible oil worldwide. This plant grows in the southern part of China and Thailand^10^. Camellia seed oil is currently used as edible oil, especially in Asia. Camellia seed oil has an abundance of nutrients, such as unsaturated fatty acids and vitamins^11^. In addition to its advances as a dietary oil, this oil also has advantages for the skin and contains oleic acid, palmitic acid, linoleic acid, and α-tocopherol^12^. These substances make this oil a powerful moisturizer that protects and repairs the skin barrier. Moreover, camellia seed oil has been reported to have antioxidant, antimicrobial, anti-inflammatory, and antimelanogenesis properties^13–15^. Concerning the sensorial effect, camellia seed oil is a very light oil that can absorb rapidly. These factors make the camellia seed oil an excellent candidate for topical products.

Forming an emulsion into liquid crystals can depend on various factors, such as the emulsion composition and temperature^16, 17^. Changing the types and concentrations of surfactants could be another factor that affects the liquid crystals^6^. Many chemical suppliers have developed surfactants, such as alkyl glycosides, polyglycerol esters, and phosphates, to prepare emulsions with liquid crystal formations^7^. Olivem 1000 is an oil-in-water emulsifier derived from olive oil. This product is composed of cetearyl olivate and sorbitan olivate^18^. Polyaquol-2W is another oil-in-water emulsifier composed of polyglyceryl-2-stearate, glyceryl stearate, and stearyl alcohol^19^. Nikkomulese LC is a blend of emulsifiers composed of lipids, fatty alcohols, lecithin, phytosterol, and emulsifiers^20^. Nikkomulese LC can provide a long-lasting moisturizing effect. Lecinol S-10 is composed of hydrogenated lecithin derived from soy which can provide a moisturizing effect while enhancing the skin barrier function. These four emulsifiers can be purchased and are currently on the market.

Currently, most studies related to liquid crystals mainly focus on the nanostructure of liquid crystal systems, such as molecular assemblies, liquid crystals characterization, and stability, without comparing the efficacy of the liquid crystal formulations clinically. In this study, liquid crystal emulsions made from camellia seed oil with four different surfactants were created. The liquid crystal emulsions preparation, thorough characterization, and formulation stability during storage were investigated. The possibility of skin irritation by the produced formulations was tested using a 3-dimensional in vitro reconstructed human epidermis. Moreover, TEWL and skin hydration of the volunteers after liquid crystal application were studied to establish the efficacy of the preparation.

## Materials and methods

### Materials

Since this study aimed to investigate the emulsifier type on liquid crystals, oil and other ingredients in the formulation were kept constant. The oil phase mainly comprised *Camellia oleifera* seed oil (Tea oil plant and other oil crops research and development center, The Chaipattana Foundation, Thailand, Lot. No. PRFA1-01070929). *Camellia oleifera* seed oil was characterized according to Supplementary Table 1. Moreover, the fatty acid composition was fully elucidated (Supplementary Table 2). Four types of emulsifier systems were selected, which were Olivem 1000 (lot no. UG1145, B&T S.r.l. Biologic & Technology, Italy), Polyaquol-2W (lot no. BB5P2F3, Innovacos, USA), Nikkomulese LC (lot no. 739913, Nikko Chemicals Co., Ltd., Japan) and Lecinol S-10 (hydrogenated lecithin, 25–35% phosphatidylcholine content, lot no. 7205, Nikko Chemicals Co., Let., Japan) mixed with Tween 80 (polyoxyethylene^20^ sorbitan monooleate), lot no. 603862, Namsiang company limited). Olivem 1000 is a mixture between cetearyl olivate and sorbitan olivate. Polyaquol-2W is composed of glyceryl stearate, polyglycerol-2 stearate, and stearyl alcohol. Nikkomulese LC is a mixture of various substances: cetyl alcohol, stearyl alcohol, behenyl alcohol, phytosterol, glyceryl stearate, caprylic/capric triglyceride, hydrogenated lecithin, and PEG-20 phytosterol. Tradename, INCI name of emulsifiers, and the amount used in each formulation are reported in Table 1. The water used in the formulation is distilled water (pH 5–5.5).

**Table 1:** Trade name, International Nomenclature Cosmetic Ingredient (INCI) name, and the amount used of four selected emulsifiers.

Formulation	Trade name	INCI name	Amount used (%w/w)
Formulation A	Olivem 1000	Cetearyl olivate Sorbitan olivate	5.00
Formulation B	Polyaquol-2W	Glyceryl stearate Polyglycerol-2 stearate Stearyl alcohol	5.00
Formulation C	Nikkomuleus LC	Cetyl alcohol Stearyl alcohol Behenyl alcohol Phytostearol Glyceryl stearate Caprylic/capric triglyceride Hydrogenated lecithin PEG-20 phytosterol	5.00
Formulation D	Lecinol S-10 + Tween 80	Hydrogenated lecithin Polyoxyethylene^20^ sorbitan monooleate	5.00

### Emulsion formulation

In this study, four types of o/w emulsions were formulated: Formulation A (Olivem 1000), B (Polyaquol-2W), C (Nikkomulese LC), and D (Lecinol S-10 with Tween 80). The oil phase was mainly composed of camellia seed oil (10% w/w) with a small amount of tocopheryl acetate (0.5% w/w) as an emollient and antioxidant. Four o/w liquid crystal emulsions were prepared using Olivem 1000, Polyaquol-2W, Nikkomuleus LC, or a mixture of Lecinol S-10 and Tween 80 at a concentration of 5%w/w. The water phase contained propylene glycol and glycerin as humectants, sodium polyacrylate as a rheological modifier, Phenostat as a preservative, and disodium EDTA, which acted as a chelating agent to increase the stability of the formulation. The compositions of each formulation are shown in Table 2.

**Table 2:** Composition of *Camellia Oleifera* seed oil cream.

Part	No	Ingredients	Amount used (%w/w)
1	1	Distilled water	76.45–77.25^a^
	2	Disodium EDTA	0.05
	3	Glycerin	3.00
	4	Propylene glycol	2.00
2	5	Tocopheryl acetate	0.50
	6	*Camellia Oleifera* seed oil	10.00
	7	Emulsifiers^b^	5.00
3	8	Phenostat (caprylhydroxamic acid (and) phenoxyethanol (and) methylpropanediol)	1.00
4	9	Sodium polyacrylate	0.2–1.0^c^
	10	Glycerin	1.00

^a^The volume of distilled water varied according to the sodium polyacrylate in each formulation.

^b^There were four types of emulsifiers which are Olivem 1000, Polyaquol-2W, Nikkomuleus LC, and Lecinol S-10 + Tween 80 (refer to Table 1).

^c^Formulation A, B and C contained 0.20%w/w sodium polyacrylate. Formulation D contained 1.00%w/w sodium polyacrylate.

Emulsions were prepared as follows: first, the water phase (Part 1) was heated up to 80–85 °C. The oil phase (Part 2) was heated up to 75–80 °C. Then, the oil phase was poured into the water phase and homogenized at 4000 rpm for 10 min using an RS-HGM 15720 homogenizer (Rising source and supply, Thailand) to form the emulsion. The ratio of the oil phase to the water phase was approximately 1:4. After the o/w emulsion was congealed (cooling to below 40 °C), Part 3 and Part 4 were added and mixed using the dispersing head. In Part 4, sodium polyacrylate was previously dispersed in glycerin. Further experiments were conducted at least 24 h after the o/w emulsion was prepared. All ingredients are listed in Table 2.

### Macroscopic and microscopic evaluation of liquid crystals in each formulation

#### Polarized optical microscopy

Liquid crystal formation in the formulation was investigated using polarized optical microscope (Light microscope, DM2000 LED, Leica Microsystems CMS Gmbh, Germany) to observe the Maltese cross. The emulsion was observed without sample dilution with a 20 × lens. Images were taken under bright field and polarized light to observe the liquid crystal phases.

#### Wide angle X-ray scattering (WAXS)

WAXS was conducted to observe the liquid crystal system occurrence by detecting the crystallinity. Each formulation (1 g) was blended with 300 mg of locust bean gum (Ingredient Center Co., Ltd, Thailand). The sample was mounted on the sample cell and scanned between 3° and 40° with a scan step equal to 0.02 in continuous mode. X-ray patterns of the emulsion were obtained using an X-ray diffraction instrument (Miniflex 600, Rigaku, Japan). The interlayer distance between bilayers was calculated following Lee and Jeong^21^ using the relationship between Bragg's angle and the X-ray wavelength (λ = 1.54 Å) [where the interlayer distance = λ/(2sin*θ*), θ is the scattering angle and λ is the X-ray wavelength].

#### Small angle X-ray scattering method (SAXS)

SAXS was utilized to evaluate the liquid crystal systems in all four formulations. SAXS experiments were performed with SAXS beamline (BL1.3W: Small/Wide angle X-ray scattering—SAXS/WAXS, Synchrotron Light Research Institute (Public Organization), Thailand). The multipole wiggler insertion device was used as a synchrotron radiation source that generates high intensity photons with an optimized energy range of 6–9 keV. It can measure sample structures ranging between 1 and 100 nm. In the experiment, the temperature of the sample was controlled at 30 °C during the measurement. The X-ray energy was selected at 9 keV using a double multilayer monochromator. The SAXS intensity profiles were obtained by circularly averaging the measured 2D scattering patterns from a CCD detector (Rayonix SX165).

The SAXS intensity profiles were obtained as a function of the scattering vector, q [where $q=\left(4\pi \mathrm{sin}\theta /2\right)/\lambda$ , θ is the scattering angle, and λ is the X-ray wavelength]^22^. The scattering vector, q, was calculated and reported as the d-spacing or period size (α) using the Pseudo Voigt curve fitting function in the SAXSIT version 4.50 program.

#### Differential scanning calorimetry

The measurements were carried out using a DSC instrument (DSC3 + STAR system, Mettler Toledo, Switzerland) to investigate the interactions between the surfactant and water molecules^23^. Formulations A-D (10 mg) were precisely weighed into aluminum pans and quickly sealed to prevent water evaporation. An empty sealed pan was used as a reference. Samples were cooled with a cooling rate equal to − 5 °C/min from 20 to − 60 °C. The samples were kept at − 60 °C and then slowly heated up to 85 °C (heating rate equal to + 5 °C/min). Nitrogen with a flow of 20 mL/min was used as a purge gas.

### Physical, chemical, microbiological analysis, and stability study

#### Formulation characteristics and long-term stability

The physicochemical analysis in all formulations after 24 h of preparation was subjected to the following study: organoleptic properties, centrifugation test, viscosity, rheology profile, and pH.

##### Organoleptic properties

Organoleptic studies were carried out by observing the sensorial properties of color, odor, texture, consistency, and the emulsion homogeneity.

##### Centrifugation test

Approximately 10 g of a liquid crystal emulsion was centrifuged at 6000 rpm for 20 min (Spectrafuge 6C, Labnet International, Inc., USA) at room temperature. Any sign of nonhomogeneity, such as phase separation, was recorded.

##### Viscosity

The viscosity of emulsions was measured using HAKKE RotoVisco 1 rotational rheometer (ThermoScientific, USA) using a cone-and-plate (C35/2°Ti L, L09034). The measurement was conducted with a shear rate of 5 1/s for 300 s at 30.0 ± 0.5 °C. The viscosity was measured every 30 s and reported as an average value.

##### Rheology profile

The rheology profile of each formulation was also studied using HAKKE RotoVisco 1 rotational rheometer with cone-and-plate (C35/2°Ti L, L09034), a shear rate controlled-ramp mode. The starting shear rate was equal to 0 1/s and increased gradually to 100 1/s in a linear manner (60 s). Afterward, the shear rate was reduced from 100 1/s to 0 1/s in 60 s. The temperature of the system was set at 30.0 ± 0.5 °C.

##### pH

The pH value was identified using an Inlab viscous pH meter (Mettler Toledo, USA).

#### Microbiological evaluation

Three types of microbiological evaluations were conducted in all formulations: (1) total viable aerobic plate count, (2) microbial limit tests, part II tests for specified microorganisms, and (3) antimicrobial effectiveness tests.

1. The total viable aerobic plate count was conducted according to USP 41: Chapter 61, microbiological examination of nonsterile products: microbial enumeration tests^24^. The acceptance criterion is less than 10 cfu per g of the product .
2. The specified microorganisms were conducted according to USP 41: Chapter 61, microbiological examination of nonsterile products: tests for specified microorganisms^24^. Four types of microorganisms: *Clostridium* spp.,* Pseudomonas aeruginosa, Staphylococcus aureus,* and *Candida albicans*, were examined. The product complies with the test if colonies are not present in 1 g of a test sample.
3. Antimicrobial effectiveness testing was conducted following the USP 41: Chapter 51 antimicrobial effectiveness testing, compendial product category 2 topically used products^25^. Samples were inoculated with five different types of microorganisms, which are *Escherichia coli* (ATCC No. 8739), *Pseudomonas aeruginosa* (ATCC No. 9027), *Staphylococcus aureus* (ATCC NO. 6538), *Candida albicans* (ATCC No. 10231), and *Aspergillus brasiliensis* (ATCC No. 16404)^25^. The requirements are met if the bacterial count is not less than 2.0 log reduction from the initial count at 14 days and no increase from the 14-day count at 28 days. Moreover, there was no increase from the initial calculated count at 14 and 28 days for yeast and molds.

#### Stability study

All formulations were subjected to accelerated stability testing. After being fully characterized, the emulsions were placed in tightly closed glass jars and kept under different storage conditions, which were 4 ± 2 °C, room temperature (approximately 30 °C), and 40 ± 2 °C. The samples were analyzed for organoleptic properties, viscosity, and pH at 14 days, and 1, 2, 3, and 6 months.

### In vitro skin irritation

All formulations were assessed for skin irritation potential following OECD guidelines for the testing of chemicals (Test guideline No. 439: In vitro skin irritation: Reconstructed human epidermis test method) with some modifications^26^. The reconstructed human epidermis (RhE) used in this experiment was an Episkin small model (Episkin, France). Upon receieved, RhE was incubated with a prewarmed maintenance medium in a 12-well plate for 24 h at 37 °C, with 5% CO_2_. The selected condition at 37 °C with 5% CO_2_ was used to match physiologic conditions in the body and to control a stable physiological pH. The skin was then exposed to 10 µL of test sample for 24 h. The skin was gently rinsed with 1 × phosphate-buffered saline (PBS) to remove the excess sample from the RhE surface, transferred to a new 12-well plate, and incubated with maintenance medium for 42 h. At the end of the experiment, the skin was rinsed with 1xPBS and incubated with 0.3 mg/mL MTT [3-(4,5-dimethylthiazol-2-yl)-2,5-diphenyltetrazolium bromide, Thaizolyl blue] for 3 h. The formazan crystals were extracted using acidified isopropanol, 500 µL per well. The supernatant was collected, and the absorbance was measured at 570 nm via a spectrophotometer (Microplate reader, Model CLARIOstar, BMG Labtech, Germany). The negative control was cells that were treated with 10 µL of 1 × PBS. The positive control was cells that were treated with 10 µL of 5% w/v sodium lauryl sulfate (SLS). The percentage of cell viability was calculated using the following equation:

$$\% \; cell \; viability= \frac{{Absorbance}_{(test \; sample)}}{{Absorbance}_{(PBS-treated)}}\times 100$$

The formulation that gives the percentage of cell viability above 50 can be considered as non-irritants in accordance with UN GHS "No category". However, if the formulation gives a percentage of cell viability below or equal to 50, that substance is considered irritant to the skin in accordance with UN GHS "Category 2"^26^.

### In vivo analysis: measuring transepidermal water loss (TEWL) and skin hydration

#### Study participants

The TEWL and skin hydration studies were approved by the Faculty of Dentistry/Faculty of Pharmacy, Mahidol University Institutional Review Board (MU-DT/PY/IRB, Bangkok, Thailand) with a certificate of approval number of COA.No.MU-DT/PY-IRB2019/049.3107). The volunteers were given a sufficient explanation of the study protocol and provided written informed consent. All procedures were performed in accordance with the International Conference on Harmonization Good Clinical practice guidelines.

The study was composed of 22 volunteers. Eligible volunteers included healthy men and women aged between 20 and 60 years who: were free of any dermatological or systemic disorder that would interfere with the results; were free from any wound, scratch, tattoo, or marks on the volar forearms; were not taking any medication that would interfere with the results such as steroids and antihistamines for at least 14 days prior to the experiment; were available for the study; and gave written informed consent. Exclusion criteria were individuals: with a history of allergy to *Camellia oleifera* seed oil or cosmetics in general; and females who indicated that they were pregnant or nursing an infant.

#### Measurement of TEWL and skin hydration

To precondition the test sites (volar forearms), volunteers were required to abstain from using any cleaning or skin care products on the test area for 3 days before and throughout the experiment. Volunteers were allowed to use only the commercially available mild liquid surfactant (Babi mild Ultra mild Bioganik, OsotspaCorporate, Thailand) that was provided. All volunteers were requested to visit the laboratory at least 30 min before the experimental sessions. All volunteers were asked to stay in a closed environment with a controlled temperature (25 ± 2 °C) and humidity (50 ± 5% RH) to acclimatize their skin before the experiment. Volar forearms of volunteers were marked. Transepidermal water loss (TEWL) and relative water content (or skin moisture) in the stratum corneum were quantified using a VapoMeter (Delfin Technologies, Finland) and Moisturemeter SC (Delfin Technologies, Finland), respectively. Afterward, all volunteers were required to wash their forearms with 10%w/v sodium lauryl sulfate (10% SLS) 4 times (2 min per time), clean the area with tap water, and wipe gently with a paper towel^27, 28^. The areas were left dry for 15–30 min before measuring the TEWL and the skin hydration.

The test product (Formulations A, B, C, and D) was applied at the test site (5 × 5 cm) with a volume equal to 0.1 mL by finger cot. TEWL and relative water content were measured in triplicate after 0.5, 1, 2, 4, 6, 8, and 10 h of application. Moreover, volunteers were required to remain in the environmentally-controlled room throughout the study.

### Statistical analysis

Data are expressed as the mean ± SEM. In vitro skin irritation was analyzed using a Kruskal–Wallis test with Dunn's multiple comparisons to compare the mean rank of each sample with the control group (PBS). The TEWL and the skin hydration values in the clinical trial were analyzed using repeated measures two-way ANOVA with Tukey comparisons to compare the mean of each sample at the same time point treated. All statistical tests were performed using GraphPad Prism version 7.00 for Windows (GraphPad Software, USA, www.graphpad.com). A *p*-value less than 0.05 was considered significant.

## Results

### *Camellia oleifera* seed oil characterization

This study aimed to observe the liquid crystal characteristics of different formulations prepared with different surfactants from the molecular level to clinical applications. The o/w emulsions were formulated with *Camellia oleifera* seed oil as the main ingredient in the oil phase (10% w/w). Before use, the camellia seed oil was fully characterized and found to have low rancidity, high quality, and a high smoke point suitable for hot process preparation (Supplementary Fig. 1). The fatty acid composition of the camellia seed oil was similar to the work previously reported by Zeng and Endo^12^. It contains various saturated and unsaturated fatty acids beneficial to the skin, such as palmitic acid, stearic acid, oleic acid, and linoleic acid (Supplementary Table 2). The fatty acids mentioned above are the main ingredients of essential fatty acids in the human epidermis^29^. These characteristic properties make camellia seed oil a good candidate as the main ingredient in the oil phase of o/w emulsion.

### Macro-and microscopic evaluation of the formulations

**Figure 1:**
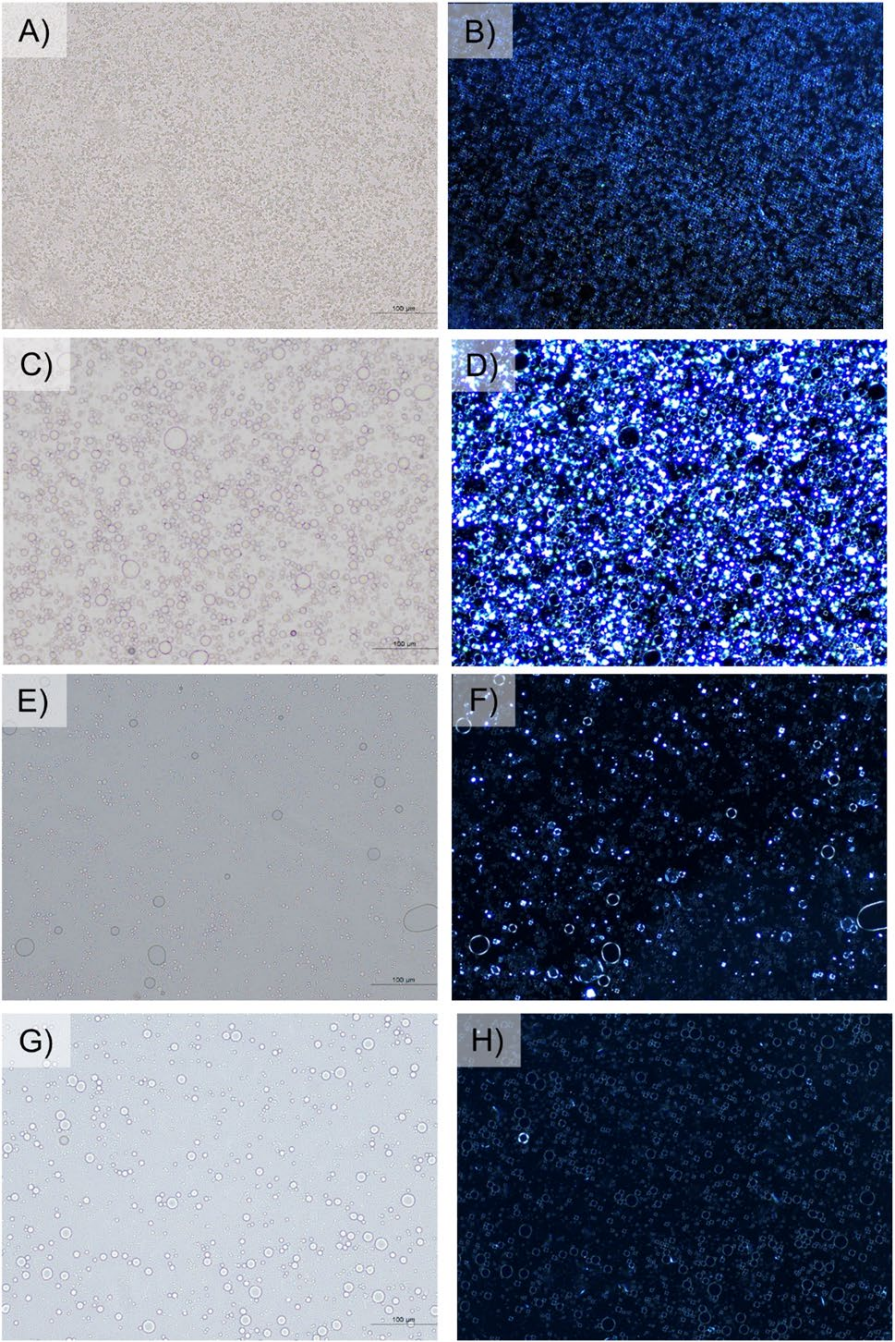
Optical micrograph under bright field (**A**,**C**,**E**,**G**) and polarized light (**B**,**D**,**F**,**H**) of Formulation A: Olivem 1000 (**A**,**B**), Formulation B: Polyaquol 2W (**C**,**D**), Formulation C: Nikkomuleus LC (**E**,**F**), and Formulation D: Lecinol S-10 + Tween 80 (**G**,**H**). Scale bar represents 100 μm.

Liquid crystals are mesophases or intermediate states of matter that exist between an isotropic liquid and a solid crystal. Because of this, liquid crystals show positional order along some directions as well as orientational order. These substances exhibit the remarkable optical characteristics of solid crystals and flow like isotropic fluids. Since the formation of liquid crystalline phases is an anisometric molecular shape, the liquid crystal emulsion possesses a degree of anisotropy. These liquid crystal systems give rise to birefringence that can be observed under a polarization microscope. According to Fig. 1, all formulations clearly showed birefringence. Based on the molecular assemblies of amphiphilic molecules, four typical liquid crystalline structures, including cubic, hexagonal, lamellar, and reversed hexagonal shapes, can be formed^30^. The formation of each structure depends on the numerical value of the critical packing parameter (CPP), which is associated with the cross-sectional area of the hydrophilic group, the extended length of the hydrophobic chain, and the volume of the hydrophobic group. The cubic liquid crystals do not show optical anisotropy due to the symmetric configuration^31^. As a result, the birefringence under a crossed-polarized light microscope cannot be observed. In this study, all formulations showed birefringence, indicating structures other than cubic, possibly a lamellar, since the birefringence observed was likely in Maltese crosses^32, 33^. However, other techniques should be applied to identify the actual molecular assembly of the liquid crystal structures, e.g., WAXS and SAXS.

**Figure 2:**
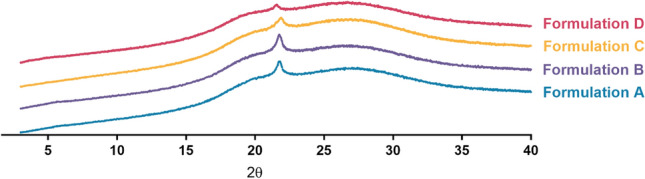
Wide angle X-ray scattering spectroscopy (WAXS) patterns of Formulation A: Olivem1000, Formulation B: Polyaquol 2W, Formulation C: Nikkomulese LC, and Formulation D: Lecinol S-10 + Tween 80 according to 2θ values.

WAXS is one of the fundamental experimental techniques for evaluating the atomic arrangement of materials. A material with a crystalline structure can reflect X-rays. In our study, all formulations showed a distinct peak at 2*θ* of 22° in all formulations tested, as demonstrated in Fig. 2. The appearance peak in the X-ray diffractogram indicates the formation of a liquid crystalline emulsion. Moreover, from this diffraction peak, the interlayer distance of the lamellar was equal to 4.04 Å. According to de Oliveira et al., when systems made of water and lipids were subjected to X-ray diffraction studies, systems with rigid carbon chains showed a sharper peak at 4.1 Å, while systems with chains structured as liquid crystals showed a broad peak at 4.5 Å. However, it was reported that emulsions containing higher than 47% water present a peak at 4.04 Å, indicating the presence of lipid bilayers in the gel phase^34^.

**Figure 3:**
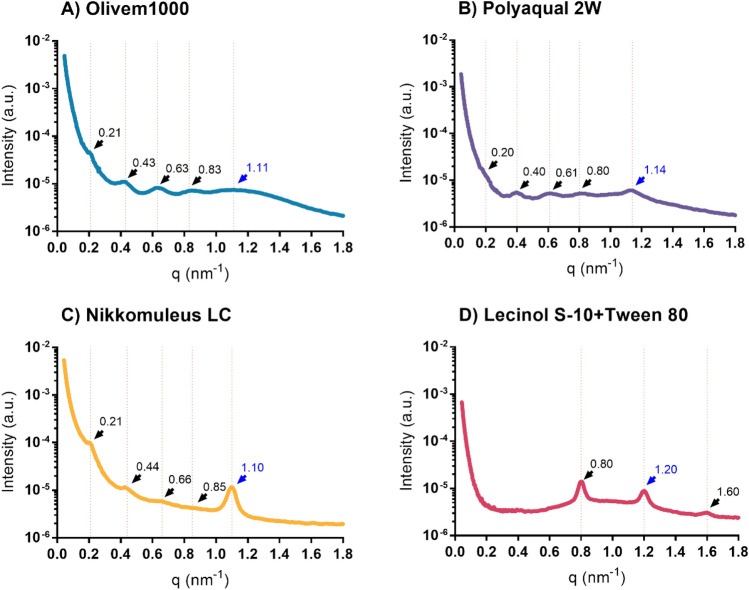
Small-angle X-ray diffraction pattern and fits of the Formulation A: Olivem1000 (**A**), Formulation B: Polyaquol 2W (**B**), Formulation C: Nikkomulese LC (**C**), and Formulation D: Lecinol S-10 + Tween 80 (**D**) measured at 30 °C. The measured data were shown using log scale for the intensity (Y-axis). Peak positions of the lamellar liquid crystal structures are shown in black arrows. Peak positions of the other structure are shown in blue arrows.

To obtain more information about the internal structure of liquid crystalline emulsions, SAXS was performed. SAXS is the most appropriate technique for determining the types of liquid crystalline structures using the Bragg peak positions in the diffraction pattern^30^. These peaks in the SAXS pattern are related to the period of the periodic structure (*d*) of the liquid crystal by *d* = 2nπ/*q*_peak_ where n is the diffraction order. By considering the multiple peak positions obtained in the SAXS pattern, the liquid crystal type can be identified from the ratio of the peak positions^35^. The SAXS patterns of all formulations are shown in Fig. 3. The peak position, peak ratio, and average period size were measured (Table 3). All formulations showed Bragg diffraction peaks (or the spacing ratio) following the relationship of 1/2/3 (q1:q2:q3), indicating the formation of liquid crystals oriented in the lamellar form (black arrows, Fig. 3). The average period size was calculated by averaging the period values obtained from all three peak orders (q1, q2, and q3). However, in all formulations tested, there was another peak that came from a different periodic structure (blue arrows, Fig. 3). In addition to identifying the liquid crystal structures, the actual peak positions obtained from SAXS can provide information on the lamellar repeat distance (d-spacing)^6^. According to Table 3, Formulations A, B, and C had d-spacings in approximately the same range (29.20–31.17 nm), while Formulation D showed a much smaller range (7.86 nm). This result indicates that the unit cell dimension (of the surfactants and water) of Formulation D is tighter than that of the other formulations. Considering all results from the polarization microscope, WAXS, and SAXS, it can be concluded that the lamellar liquid crystal emulsion was observed in all formulations.

**Table 3:** Peak position, peak ratio (or the spacing ratio), and the d-spacing (or the period size (α)) of the lamellar liquid crystal structures in the 4 formulations tested.

Formulation	Peak order	Peak position (q, nm^−1^)	Peak ratio	d-spacing (α, nm)
Formulation A	1	0.21	1.00	29.87
	2	0.43	2.03	
	3	0.63	3.02	
	4	0.83	3.95	
Formulation B	1	0.20	1.00	31.17
	2	0.40	1.96	
	3	0.61	2.98	
	4	0.80	3.90	
Formulation C	1	0.21	1.00	29.20
	2	0.44	2.09	
	3	0.66	3.17	
	4	0.85	4.04	
Formulation D	1	0.80	1.00	7.86
	3	1.60	1.99	

**Figure 4:**
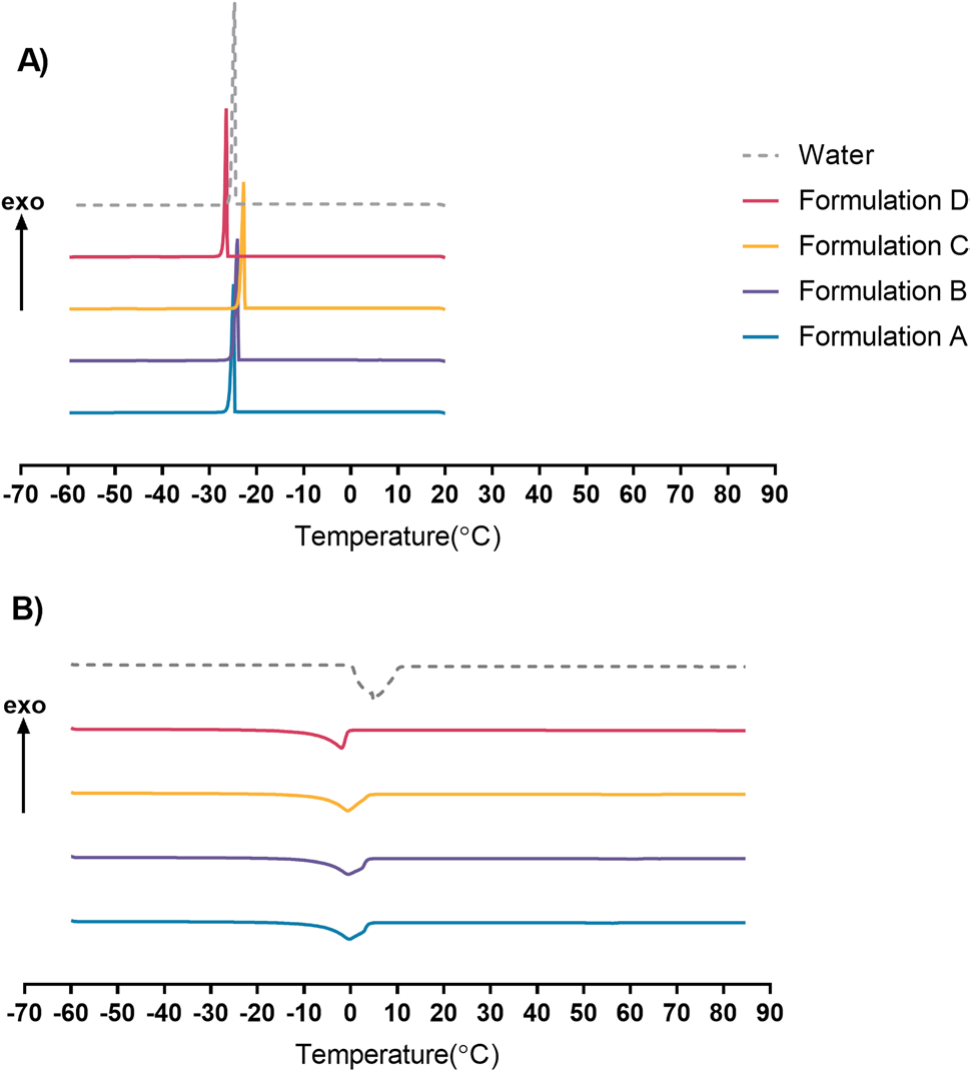
DSC cooling curves (**A**) and heating curves (**B**) of the Formulation A: Olivem1000 (**A**, blue), Formulation B: Polyaquol 2W (**B**, purple), Formulation C: Nikkomulese LC (**C**, yellow), and Formulation D: Lecinol S-10 + Tween 80 (**D**, pink), and distilled water (water, grey).

DSC was conducted to analyze the state of water in the liquid crystal samples (Fig. 4). Distilled water or bulk water was used as a reference. The exothermic and endothermic peaks obtained from each formulation are shown in Table 4. In the cooling curve (Fig. 4A), distilled water showed a large and sharp exothermic peak at approximately − 24.67 °C. A wide endothermic peak was found in the water heating curve, with the peak equal to 4.92 °C corresponding to the melting of pure water (Fig. 4B). According to Bonacucina et al.^36^ and Kodama et al.^37^, water in the interbilayer region that does not interact with polar head groups of surfactants shows a freezing temperature similar to the bulk free water. The freezing temperature depends on the water and surfactant content and the strength of the interaction. Samples with lower water crystallization temperatures represent strong water-surfactant interactions. Formulation D showed the cooling curve with the sharp peak at temperature lower than that of distilled water. This indicates the interaction between water and polar head groups of emulsifiers. Concerning the heating curves, all formulations showed broad endothermic peaks corresponding to ice melting from freezable interlamellar water. Endothermic peaks in all formulations shifted toward lower temperatures than distilled water (4.92 °C) which was attributed to the freezable bound water in the formulations^38^. From the above, it can be concluded that all formulations contained freezable bound water at the interlayer spacing of the liquid lamella. Among all formulations, Formulation D had the most robust water-surfactant interaction as seen by the lowest temperature of the exothermic peak (− 26.42 °C) and the lowest temperature of the endothermic peak (− 1.92 °C). This result is consistent with the SAXS results obtained from Formulation D, which showed a small repeat distance. The difference in the interaction between water and surfactant among formulations may affect skin moisturization application.

**Table 4:** DSC parameters of exothermic peaks and endothermic peaks in the 4 formulations tested.

Formulation	Exothermic peak (°C)	Endothermic peak (°C)
Formulation A	− 24.92	− 0.33
Formulation B	− 24.08	− 0.50
Formulation C	− 21.33	− 0.50
Formulation D	− 26.42	− 1.92
Water	− 24.67	4.92

### Formulation characteristics

#### Physical, chemical, and microbiological analysis

All formulations were off-white color cream with a distinct smell from *Camellia oleifera* seed oil. Formulation D was slightly yellow when compared to other formulations. The color difference was caused by Lecinol S-10, which is yellow-to-brown. Other emulsifiers, on the other hand, were white. The o/w emulsions were homogeneous and easily spread when applied onto the skin with a good sensory profile. All formulations had pH values between 5.9 and 6.5, which are suitable for skin administration^39^.

Changing the emulsifier in the system caused a drastic change in the viscosity and the rheology profile. In this study, the viscosity and the rheology profile of the formulations were both measured using the same instrument (HAKKE RotoVisco 1 rotational rheometer) with the same cone and plate. For the viscosity, the measurement was conducted with a constant shear rate of 5 1/s. Formulation B (5,145 cP) had the highest viscosity, followed by Formulation A (3,497 cP) and Formulation D (2,209 cP). Among all samples tested, Formulation C had the lowest viscosity (860 cP) (Supplementary Figure 3).

**Figure 5:**
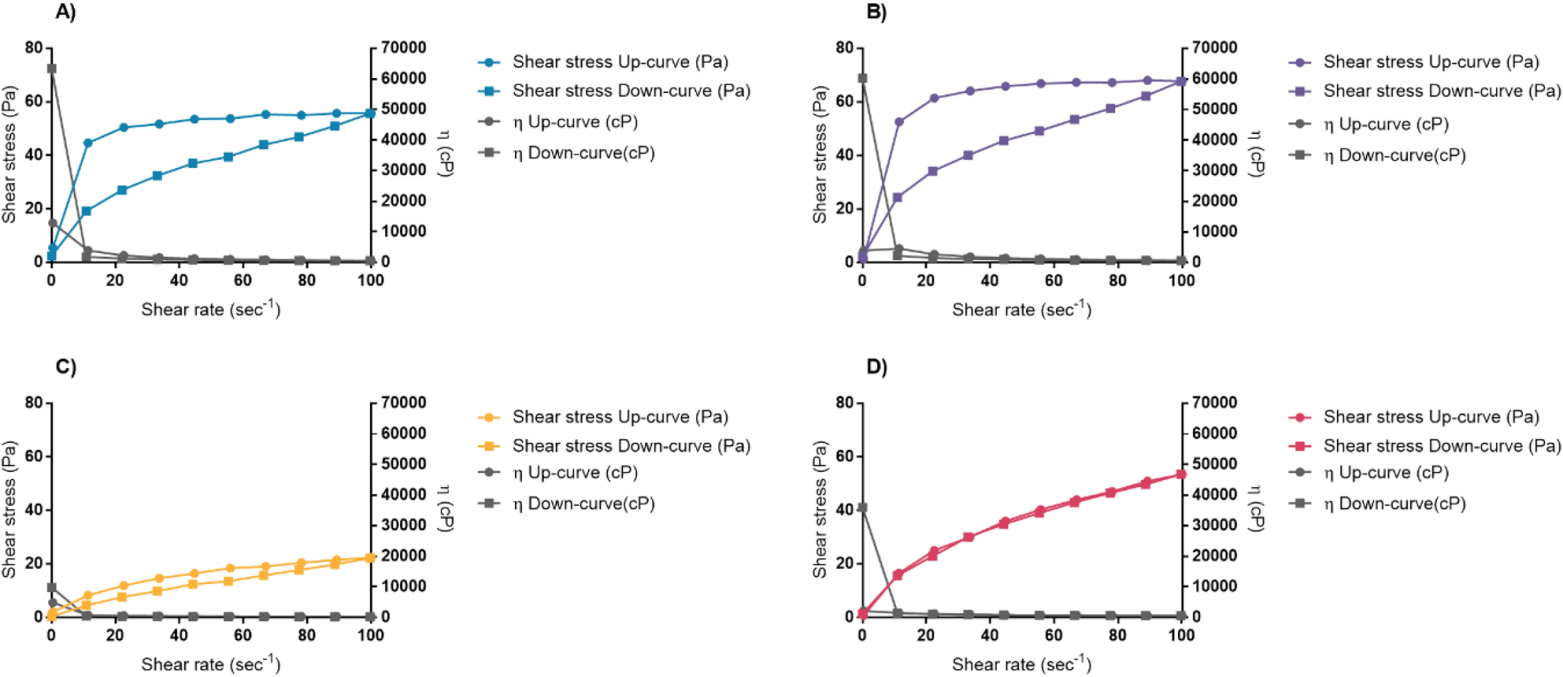
Rheology profiles obtained from Formulation A: Olivem1000 (**A**, blue), Formulation B (Polyaquol 2W) (**B**, purple), Formulation C (Nikkomulese LC) (**C**, yellow), and Formulation D (Lecinol S-10 + Tween80) (**D**, pink). Solid circle (Shear stress Up-curve) represents shear stress (in Pa) obtained by stepping up shear rate from 0 1/s to 100 1/s gradually within 60 s. Solid rectangle (Shear stress Down-curve) represents shear stress (in Pa) obtained by stepping down shear rate from 100 1/s to 0 1/s gradually within 60 s. Grey solid circle (η Up-curve) represents the apparent viscosity (in cP) at each shear rate during the stepping up shear rate. Grey rectangle (η Down-curve) represents the apparent viscosity (in cP) at each shear rate during the stepping down shear rate. X-axis represents shear rate (1/s). Left Y-axis and right Y-axis represent shear stress (Pa) and the apparent viscosity (cP), respectively.

The rheology profile of each formulation was studied using a shear rate with controlled-ramp mode, stepping up the speed from 0 1/s to 100 1/s within 60 s and stepping down the speed from 100 1/s to 0 1/s within 60 s. This condition was set to see the rheology profile and to observe the thixotropy property of the formulation. The rheology profiles obtained are shown in Fig. 5. All formulations showed pseudoplastic flow with shear-thinning properties. Moreover, thixotropy can be found in Formulations A, B, and C as seen in the form of the hysteresis loops (enclosed area by the shear stress up-curve and the shear stress down-curve). However, Formulation D had no thixotropy. The shear thinning behavior is a good characteristic of the pharmaceutical emulsion since it improves the spreadability when the emulsion is applied onto human skin in actual application conditions^40^.

All formulations passed all three types of microbiological evaluation according to USP 41—NF 36, including the total viable aerobic plate count, the specified microorganisms, and the antimicrobial effectiveness testing, indicating that Phenostat is a suitable preservative for all developed formulations.

### Formulation stability

The centrifugation test was conducted as additional testing to predict any possible instability of the formulation, especially for emulsions. After the emulsions were centrifuged at 6000 rpm for 20 min, only Formulation C separated. Other formulations were still homogeneous with no sign of creaming or phase separation (Supplementary Fig. 1). Centrifugation at high speed over a range of times can accelerate emulsion droplet migration. This method is helpful for predicting and comparing the emulsion stability between formulations. However, this method is a rough estimation of product stability^41^. The results obtained from the centrifugation process need to be used in combination with other stability studies, such as long-term stability.

**Figure 6:**
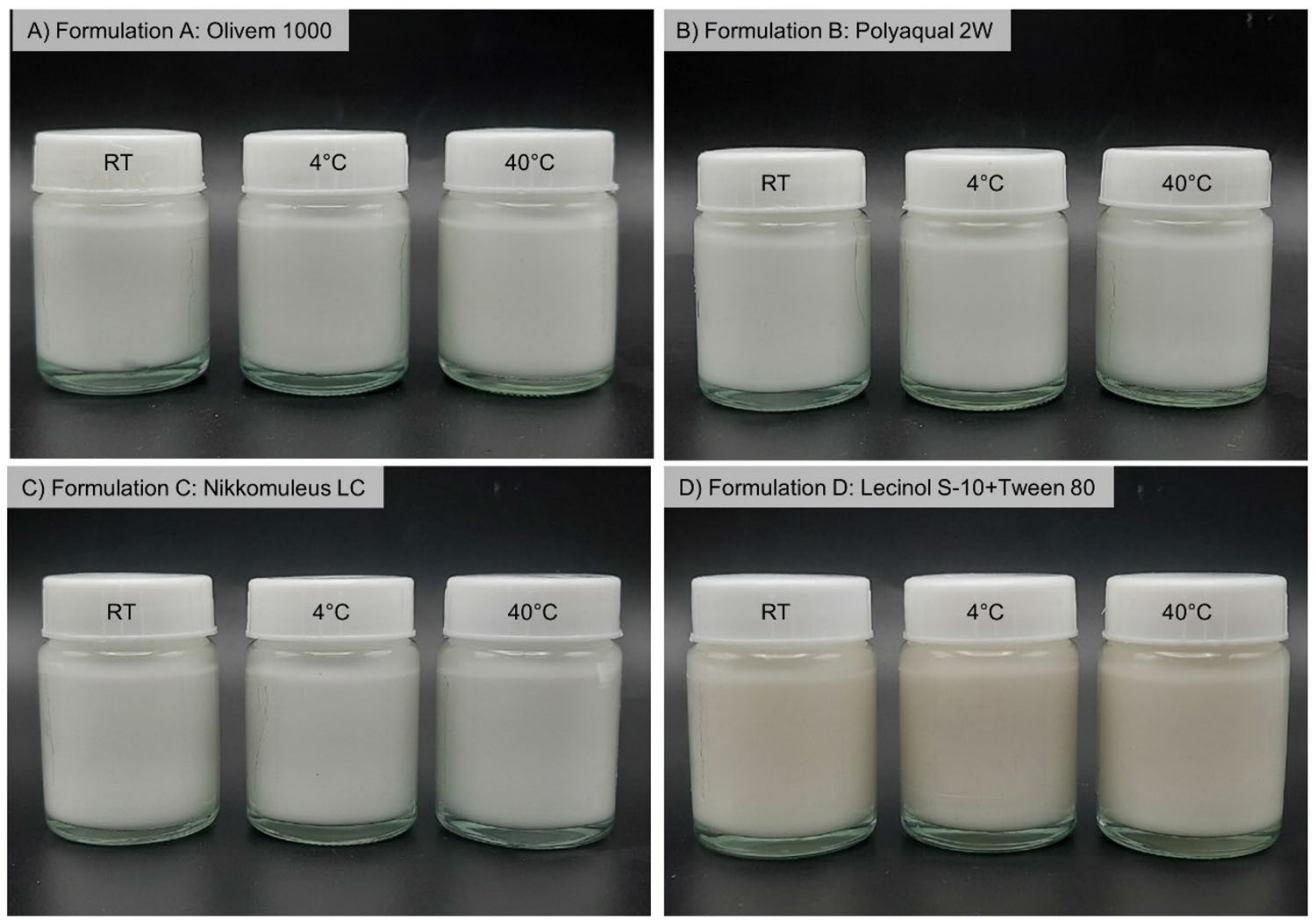
Images represent appearances from all formulations (Formulation A–D) after 6-month storage in glass bottles at room temperature (RT), 4 °C, and 40 °C.

After the formulations were fully characterized, the long-term stability (6-month storage at different temperatures) was determined. Any changes in the physicochemical properties of the emulsions (organoleptic, viscosity, and pH) in all conditions tested (RT, 4 °C, and 40 °C) were evaluated. It was found that the storage temperature did not affect the appearance of all formulations, as shown in Fig. 6. The appearance of all formulations that were kept at different temperatures was comparable. Moreover, pH values in all formulations were between 5.5 and 6.5, which were not different from the initial period (Day 0) (Supplementary Fig. 2).

Viscosity is another factor that was monitored. The viscosity in Formulation A, Formulation B, and Formulation D was constant throughout 6 months of storage in all conditions tested (Supplementary Fig. 3). However, the viscosity of Formulation C was increased when kept at 40 °C for longer than 3 months (Supplementary Fig. 3C).

Formulations A, B, and D had good stability under normal and stress conditions. They did not show any signs of phase separation after centrifugation or when kept in stress conditions for 6 months. Nevertheless, Formulation C showed signs of possible instability since it had phase separation after centrifugation. This could come from the fact that Formulation C had the lowest viscosity.

### In vitro skin irritation

**Figure 7:**
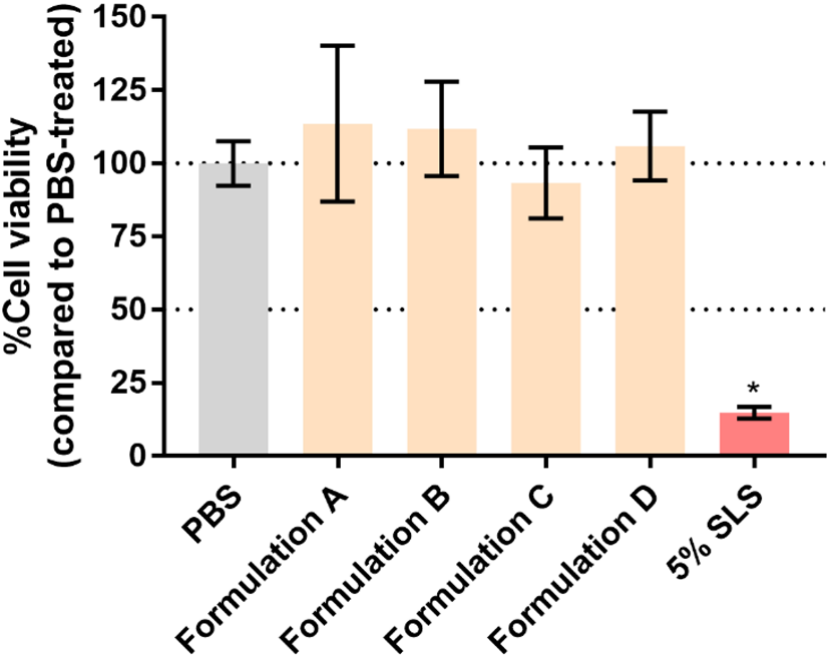
Percent cell viability of Episkin when treated with Formulation A: Olivem1000, Formulation B: Polyaquol 2W, Formulation C: Nikkomulese LC, and Formulation D: Lecinol S-10 + Tween 80 for 24 h. PBS was used as a negative control represented 100% cell viability. SLS (5%w/w) was used as a positive control. Data are expressed as mean ± SD, n = 3. Kruskal–Wallis with Dunn’s multiple comparisons test was performed. *p < 0.05.

The skin irritation potential of all formulations was tested following OECD Test Guideline No. 439 with a modification^26^. Using cell viability as a readout, this RhE model can measure the initiating events in the cascade of skin irritation, such as cell damage. This model can be used as a full replacement method for assessing the skin-irritancy potential of chemicals^42^. RhE was exposed to Formulations A–D for 24 h. Afterward, the cell viability was measured using the MTT dye. PBS was used as a negative control and represented 100% cell viability. The positive control (5% SLS) showed cell viability lower than 40% (*p* < 0.05). Cells in all samples tested had cell viability close to 100, which were classified as non-irritants (> 50%^26^) (Fig. 7). There was no significant difference between each formulation and the PBS-treated groups (*p* > 0.05). All formulations were considered non-irritants.

According to the protocol recommended by the OECD, the cells should be exposed to the formulations for only 15 min. Ma et al.^43^ recommend exposing the RhE to cosmetic products for 18 h. However, in this current experiment, the exposure time was set to 24 h to represent the actual exposure time by the consumer. The cell viability percentage in all formulations was still close to 100, although the exposure time was extended from 15 min to 24 h. This can ensure that all formulations are safe and can be used in humans.

### In vivo transepidermal water loss and skin hydration

This study enrolled 21 subjects (7 male and 14 female) with a median age of 24 years. Most of the subjects were between the ages of 20 and 25. The age distribution is shown in Supplementary Fig. 4.

**Figure 8:**
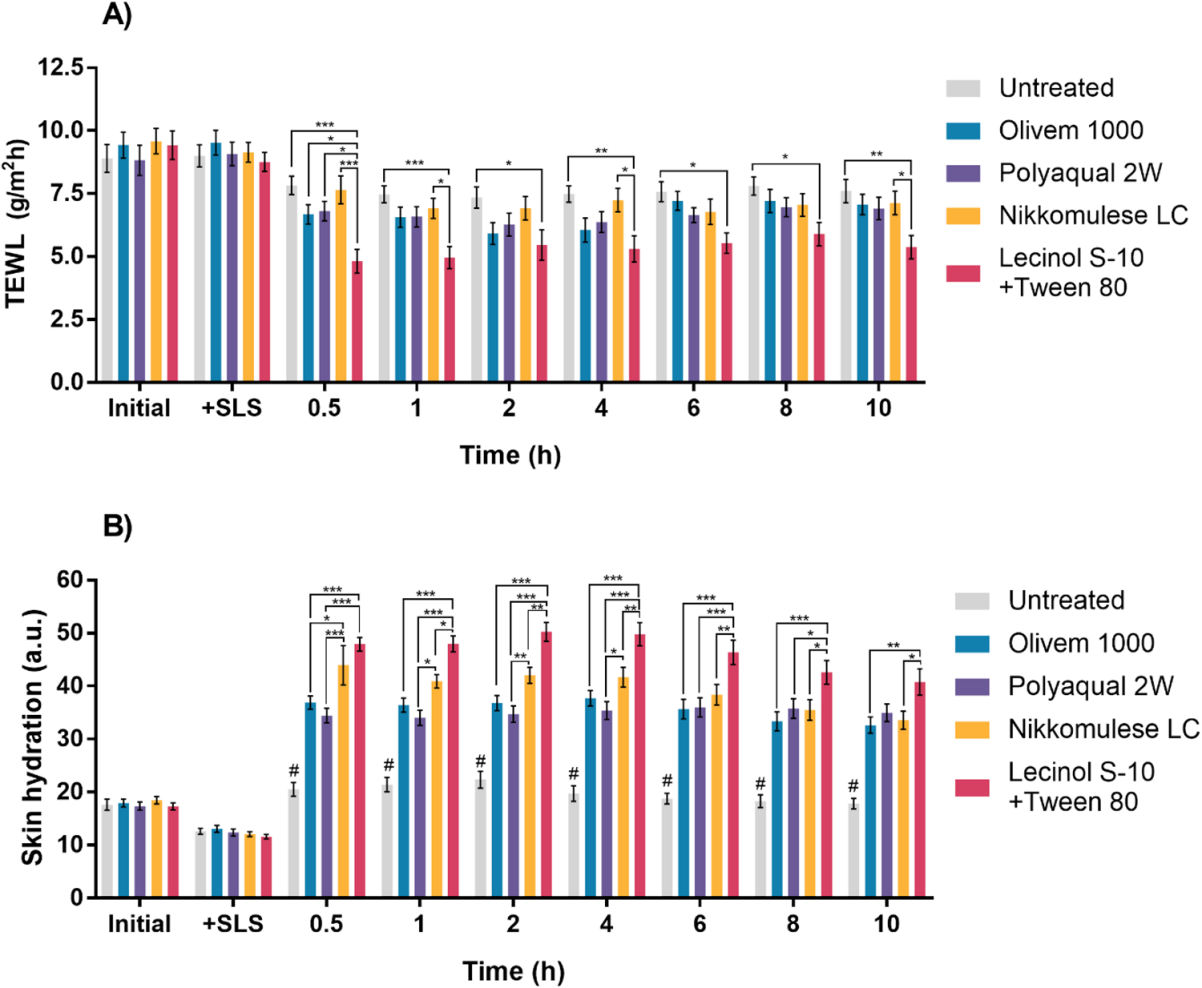
Transepidermal water loss (TEWL, g/m^2^ h) (**A**) and the skin hydration (a.u.) (**B**) obtained from 21 volunteers after a single application of Formulation A: Olivem1000, Formulation B: Polyaquol 2W, Formulation C: Nikkomulese LC, and Formulation D: Lecinol S-10 + Tween 80 for 10 h. Untreated group was the skin area where no formulation applied. Data are expressed as mean ± SEM, n = 21. Repeated measures two-way ANOVA with Tukey comparisons test was performed. *p < 0.05, **p < 0.01, ***p < 0.001. #Represents the statistical significant difference when compared untreated with each formulation.

Figure 8A shows no difference in TEWL between the initial and 10% SLS-treated groups. Cleansing the skin with 10% SLS did not elevate the TEWL. However, when the emulsions were applied, the TEWL in all formulations tested was reduced. The formulation that gave the lowest TEWL was Formulation D (Lecinol S-10 + Tween80), which showed the lowest TEWL 30 min after application which was significantly different when compared to the other groups. Formulation D is the only formulation that can significantly reduce the TEWL values for more than 10 h after cream application.

SLS was introduced to mimic the dry skin condition in volunteers. Exposing the skin to SLS can damage the skin and induce skin irritation^44, 45^. Thus, washing the skin with 10% SLS was used. Lintner et al.^27^ applied this protocol to healthy volunteers with dry skin; the mean age was 29. They found a significant increase in the TEWL values. However, in the current study, there was no change in the TEWL values after 10% SLS treatment compared to the initial values. This difference could come from the ethnic differences in skin properties and the nature of volunteer skin^46, 47^. All volunteers in our study were Asian with healthy skin.

When an emulsion is applied to the skin, the water in the emulsion progressively evaporates, increasing the ratio of oils and emulsifiers. This occurrence causes the emulsion to break down, resulting in the occlusion of oil and emulsifier on the skin’s surface. For the lamella liquid crystalline emulsion, the lamella liquid crystal structure on the skin surface was formed, mimicking the natural stratum corneum lipids^8^. Oil occlusion on the skin surface combined with the formation of lamellar liquid crystal structures can reduce water evaporation from the skin surface. This would increase the water content and decrease the TEWL. As shown in Fig. 8A, all formulations can reduce the TEWL for at least 10 h after one-time application. Among the formulations, Formulation D was the best formulation to significantly reduce TEWL in the clinical setting. From the nanostructure point of view, this phenomenon can be explained via SAXS. The calculated d-spacing of Formulation D was shorter than Formulations A, B, and C. The shorter d-spacing demonstrates the smaller or tighter lamellar structure^6^.

Another parameter, skin hydration, was also evaluated. Washing the skin with 10% SLS slightly reduced the skin hydration (Fig. 8B). After application, a significant increase in skin hydration was observed in all formulations compared to untreated skin at all time points tested (# symbol, Fig. 8B). Formulation D gave the highest skin hydration, which was significantly different from the other formulations. The result is consistent with the TEWL values. For the untreated group, washing the skin with 10% SLS reduced skin hydration. However, the skin hydration increased to the initial value after 30 min and was maintained throughout the experiment.

It is well known that the intercellular lipids of the stratum corneum form a lamellar structure composed of ceramides, cholesterols, and other amphiphilic substances. This layer acts as a skin barrier. These lipids have high moisturizing properties and reduce transepidermal water loss. Zhang and Liu et al.^7^ prepared a liquid crystal emulsion using C16-18 fatty alcohol as an emulsifier at a concentration between 2 and 8%. It was found that the water content of the skin surface was not different after applying different emulsions. However, the TEWL on the skin surface decreased with increasing C16–18 fatty alcohol content. From our experiment, the skin moisture was increased significantly after applying Formulations A–D. As previously discussed in the SAXS, all formulations formed lamella liquid crystals. These lamellar structures can entrap water molecules between the hydrophilic groups of the emulsifier molecule. As a result, the application of lamellar liquid crystalline emulsions could enhance the water content on the skin surface. Among the formulations, the application of Formulation D on the skin surface provided the highest water content. Formulation D showed the highest association between water and the hydrophilic group of emulsifiers compared to other formulations in DSC. These data correspond to the highest skin hydration in volunteers’ skin when treated with Formulation D.

The four formulations tested in this study all contained liquid crystal structures. They possessed good characteristics and were suitable to be used as a cream base for topical application to help increase skin hydration and strengthen the skin barrier. However, the formulations are not the same. Changing surfactants in the formulation resulted in nanostructure level changes. This directly affects the clinical outcomes. Among all formulations, Formulation D, composed of Lecinol S-10 with Tween 80, had a narrow molecular arrangement in the structure. Moreover, Formulation D had a high strength of freezable bound water between the ordered crystalline structures. All of the above factors make Formulation D the best formulation for reducing TEWL and increasing skin hydration.

## Conclusion

This study aims to observe the liquid crystal characteristics from different formulations prepared with different surfactants from the molecular level to clinical applications. All formulations had liquid crystal characteristics with lamellar structures. They all showed good stability under normal and accelerated conditions in long-term storage. Using the RhE as a skin model, the safety of the formulations was confirmed. In the clinical trial, all formulations reduced the TEWL and increased skin hydration immediately after application. This lasted for more than 10 h. Among all formulations tested, Formulation D, which contained Lecinol S-10 and Tween 80 as emulsifiers, showed a clear Maltese cross under the polarized microscope with a positive result for liquid crystals in WAXS and SAXS. Moreover, this formulation showed the most robust interaction between the surfactant and water molecules in the lamellar structure under DSC. The formulation itself had good stability in long-term normal and accelerated conditions. Above all, Formulation D formulated with Lecinol S-10 with Tween 80 had the best clinical result, was not irritating to the skin, and can be used as a cream base in the pharmaceutical and cosmeceutical industries.

## Supplementary Information

Supplementary Information.

## Data Availability

All data generated during and/or analyzed during this study are available from the corresponding author on reasonable request.
