# Supplementary material for: Influence of the emulsifier on nanostructure and clinical application of liquid crystalline emulsions
Source: Sci Rep. 2023 Mar 14;13:4185. doi: 10.1038/s41598-023-31329-w (PMC10015016; doi:10.1038/s41598-023-31329-w)
Supplement: Supplementary file 1 — Supplementary Information. [file 41598_2023_31329_MOESM1_ESM.pdf]

Supplementary table 1: *Camellia Oleifera* seed oil characteristic

Oil characteristic	Values obtain	Method
Acid value (mg KOH/g)	0.37	IS0 660:1996
Peroxide value (meg/kg oil)	7.79	IUPAC 2.501
Iodine Value	82.46	IUPAC 2.205 (Wijs)
Saponification Value (mgKOH/g)	190.42	IUPAC 2.202
Unsaponifiable Matter (%)	0.85	ISO 18609:2000(E)
Relative Density (25°C /water 20°C)	0.9030	AOCS Ca 10a-25
Smoke Point (°C)	230	AOCS Cc 9a-48 (1997)

Supplementary table 2: *Camellia Oleifera* seed oil fatty acid composition

Type	Fatty acid	Percent as fatty acid
Total saturated fatty acid	Palmitic acid (C16:0)	10.74
	Stearic acid (C18:0)	2.06
Total unsaturated fatty acid	Oleic acid (C18:1)	73.88
	Linoleic acid (C18:2)	12.66
	Linolenic acid (C18:3)	0.66

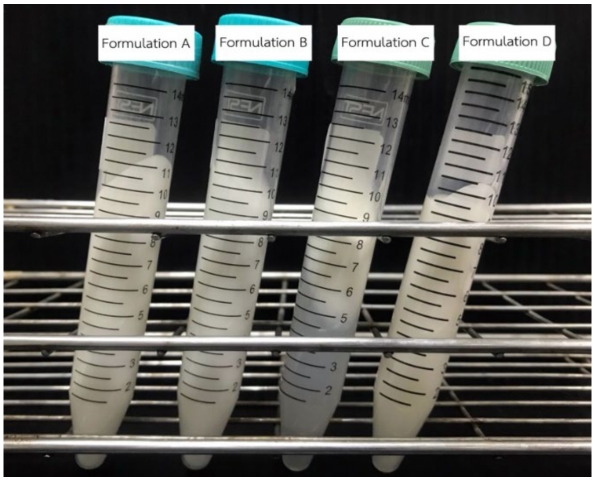

Supplementary figure 1: Image represented all formulations after being centrifuged at 6,000 rpm for 20 min. Formulation A: Olivem1000 (A), Formulation B (Polyaquol-2W) (B), Formulation C (Nikkomulese LC) (C), and Formulation D (Lecinol S-10+Tween80) (D).

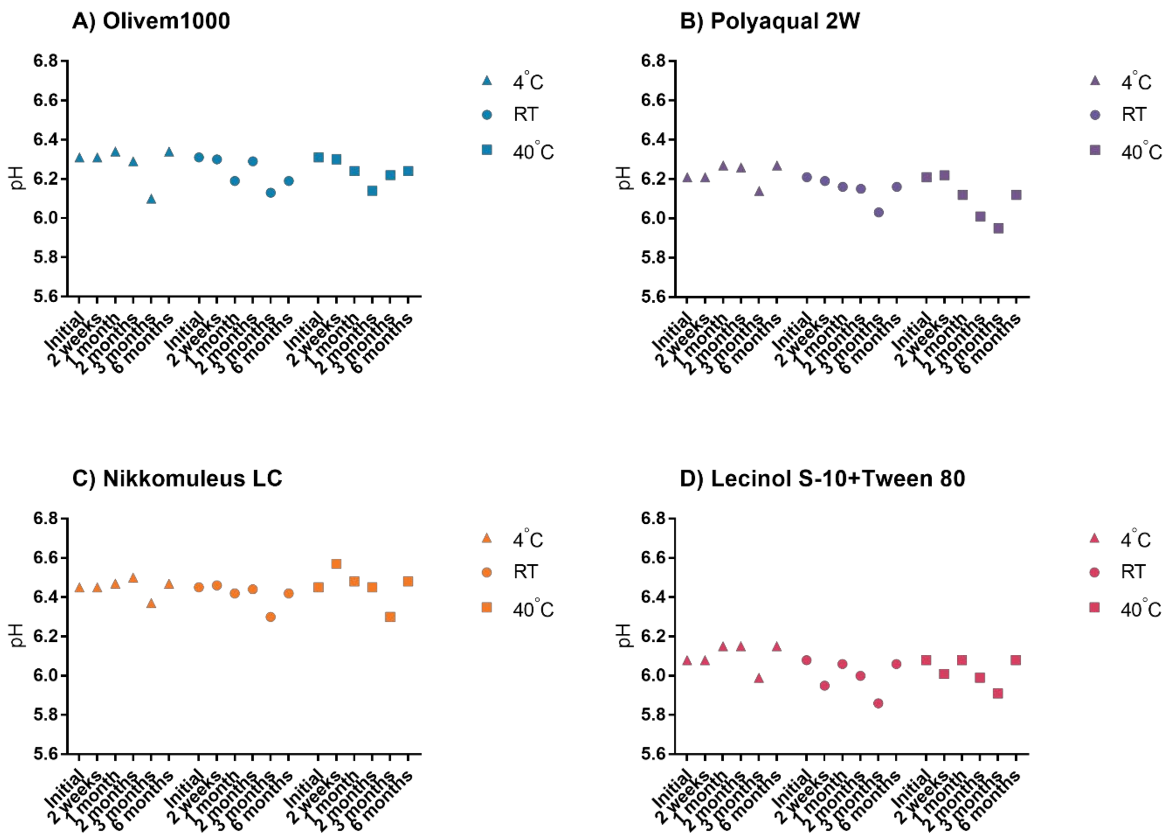

Supplementary figure 2 pH values of the Formulation A-D in three different conditions (4°C (▲), room temperature (RT, ●), and 40°C (■)) during 6 months of storage.

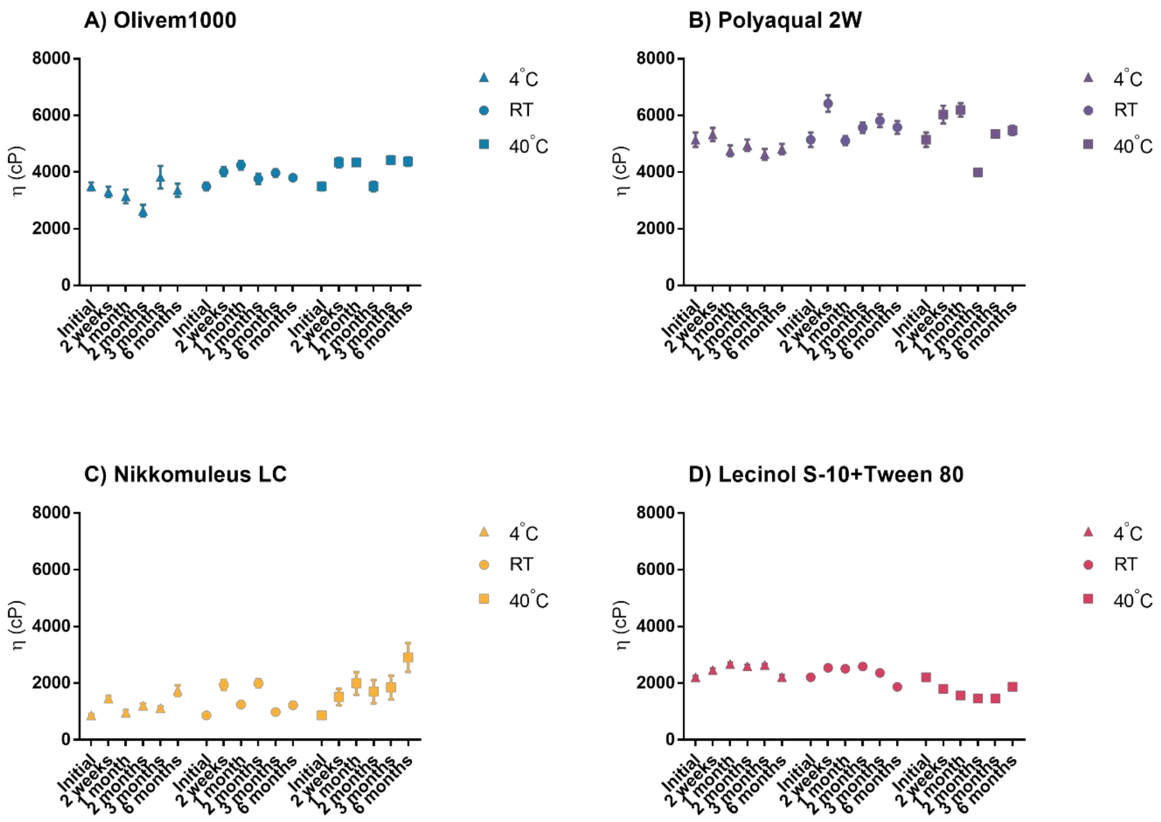

Supplementary figure 3: Viscosity (η, cP) of the Formulation A-D in three different conditions (4°C (▲), room temperature (RT, ●), and 40°C (■)) during 6 months of storage.

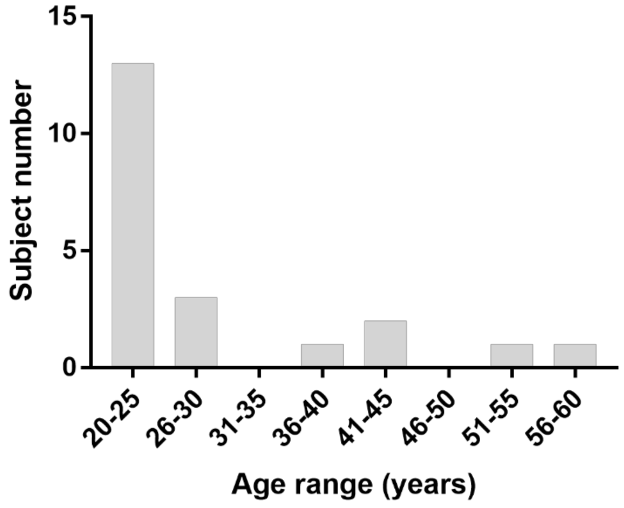

Supplementary Figure 4: The age distribution of 21 volunteers in the study. The median age is equal to 24 years.
